# Supplementary material for: HIV-1 Vpr-induced DNA damage activates NF-κB through ATM-NEMO independent of cell cycle arrest
Source: mBio. 2024 Sep 13;15(10):e00240-24. doi: 10.1128/mbio.00240-24 (PMC11481869; doi:10.1128/mbio.00240-24)
Supplement: Supplemental material — Figures S1-S6 and legends for Files S1 and S2. [file mbio.00240-24-s0003.pdf]

SUPPLEMENTAL FIGURES

Fig S1

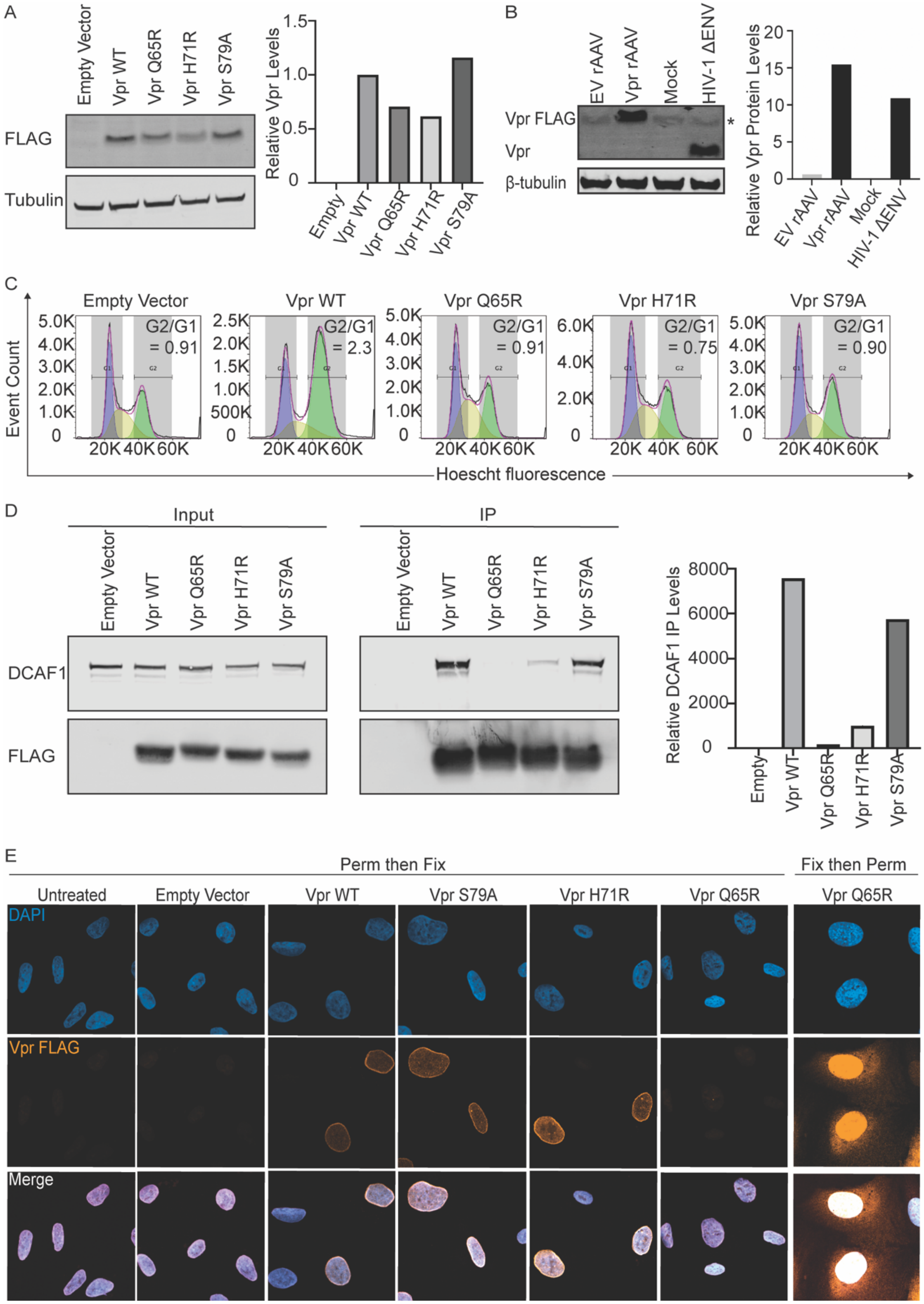

Fig S2

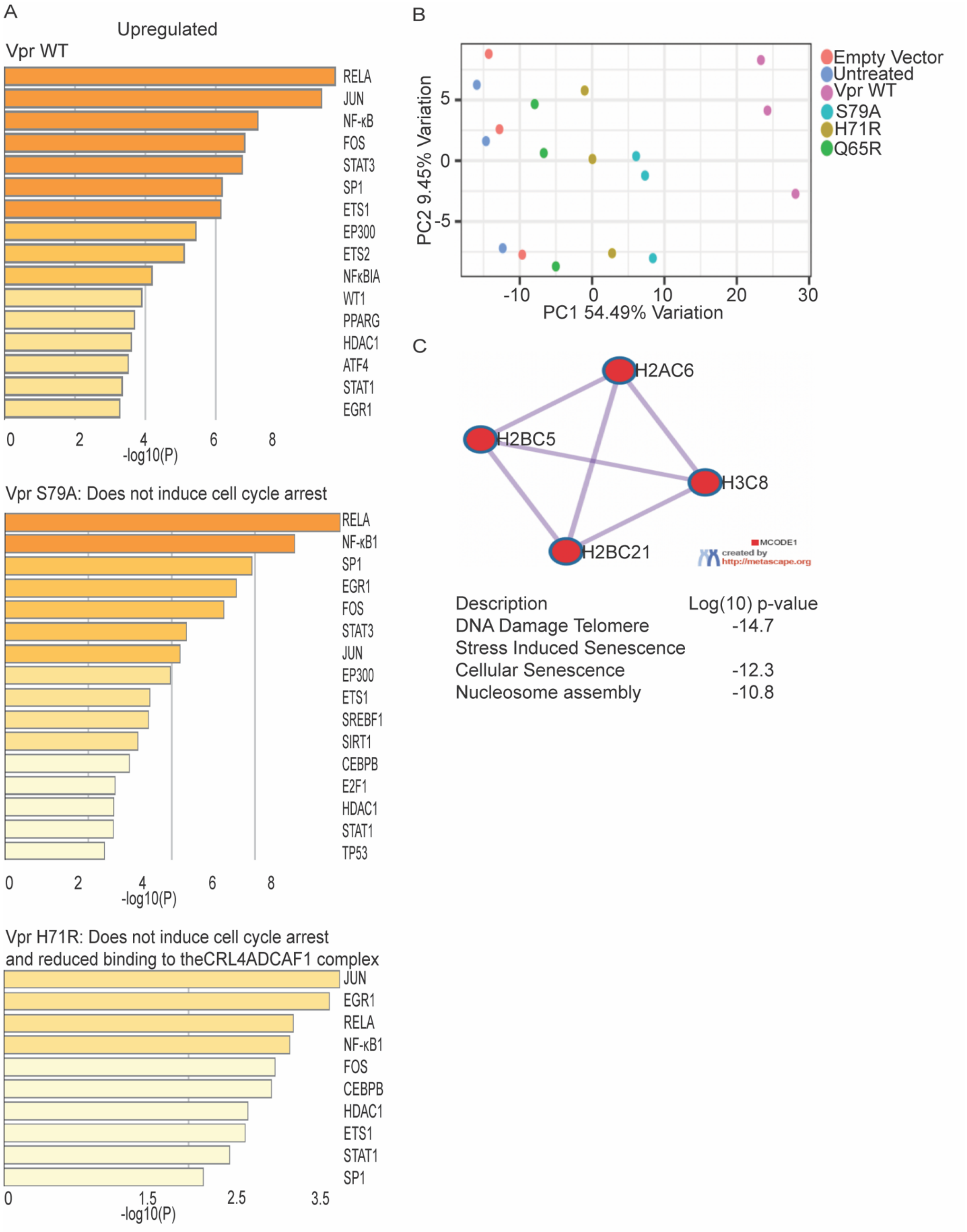
A
Upregulated
Vpr WT
RELA
JUN
NF-κB
FOS
STAT3
SP1
ETS1
EP300
ETS2
NFκBIA
WT1
PPARG
HDAC1
ATF4
STAT1
EGR1
0
2
4
6
8
-log10(P)
Vpr S79A: Does not induce cell cycle arrest
RELA
NF-κB1
SP1
EGR1
FOS
STAT3
JUN
EP300
ETS1
SREBF1
SIRT1
CEBPB
E2F1
HDAC1
STAT1
TP53
0
2
4
6
8
-log10(P)
Vpr H71R: Does not induce cell cycle arrest
and reduced binding to theCRL4ADCAF1 complex
JUN
EGR1
RELA
NF-κB1
FOS
CEBPB
HDAC1
ETS1
STAT1
SP1
0
1.5
2.5
3.5
-log10(P)
B
Empty Vector
Untreated
Vpr WT
S79A
H71R
Q65R
PC2 9.45% Variation
5
0
-5
-10
0
10
20
30
PC1 54.49% Variation
C
H2AC6
H2BC5
H3C8
H2BC21
MCODE1
created by
http://metascape.org
Description
Log(10) p-value
DNA Damage Telomere
-14.7
Stress Induced Senescence
Cellular Senescence
-12.3
Nucleosome assembly
-10.8

Fig S3

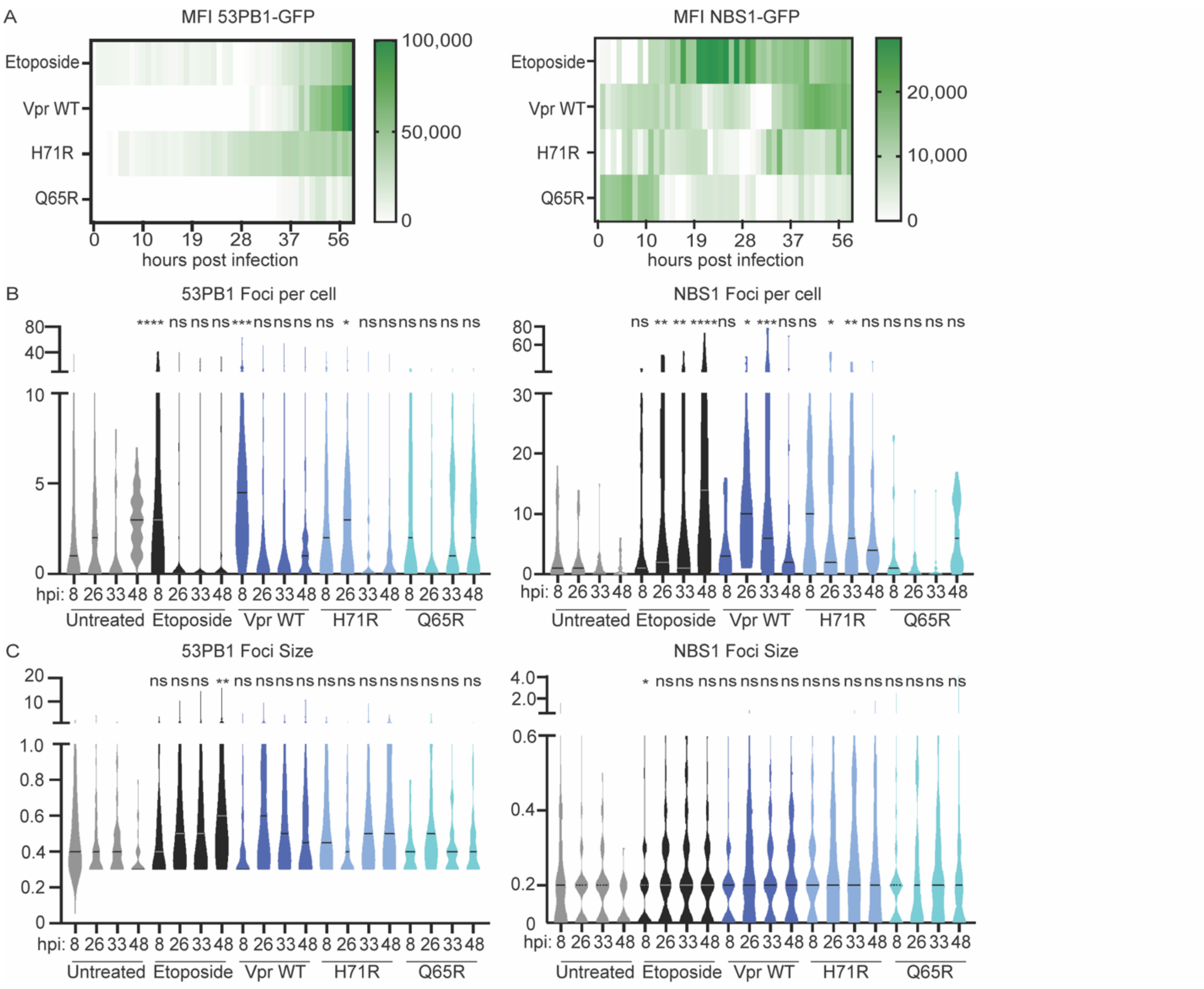
A
MFI 53PB1-GFP
MFI NBS1-GFP
Etoposide
Vpr WT
H71R
Q65R
100,000
50,000
0
20,000
10,000
0 10 19 28 37 56
hours post infection
B
53PB1 Foci per cell
NBS1 Foci per cell
hpi: 8 26 33 48 8 26 33 48 8 26 33 48 8 26 33 48 8 26 33 48
Untreated Etoposide Vpr WT H71R Q65R
C
53PB1 Foci Size
NBS1 Foci Size

Fig S4

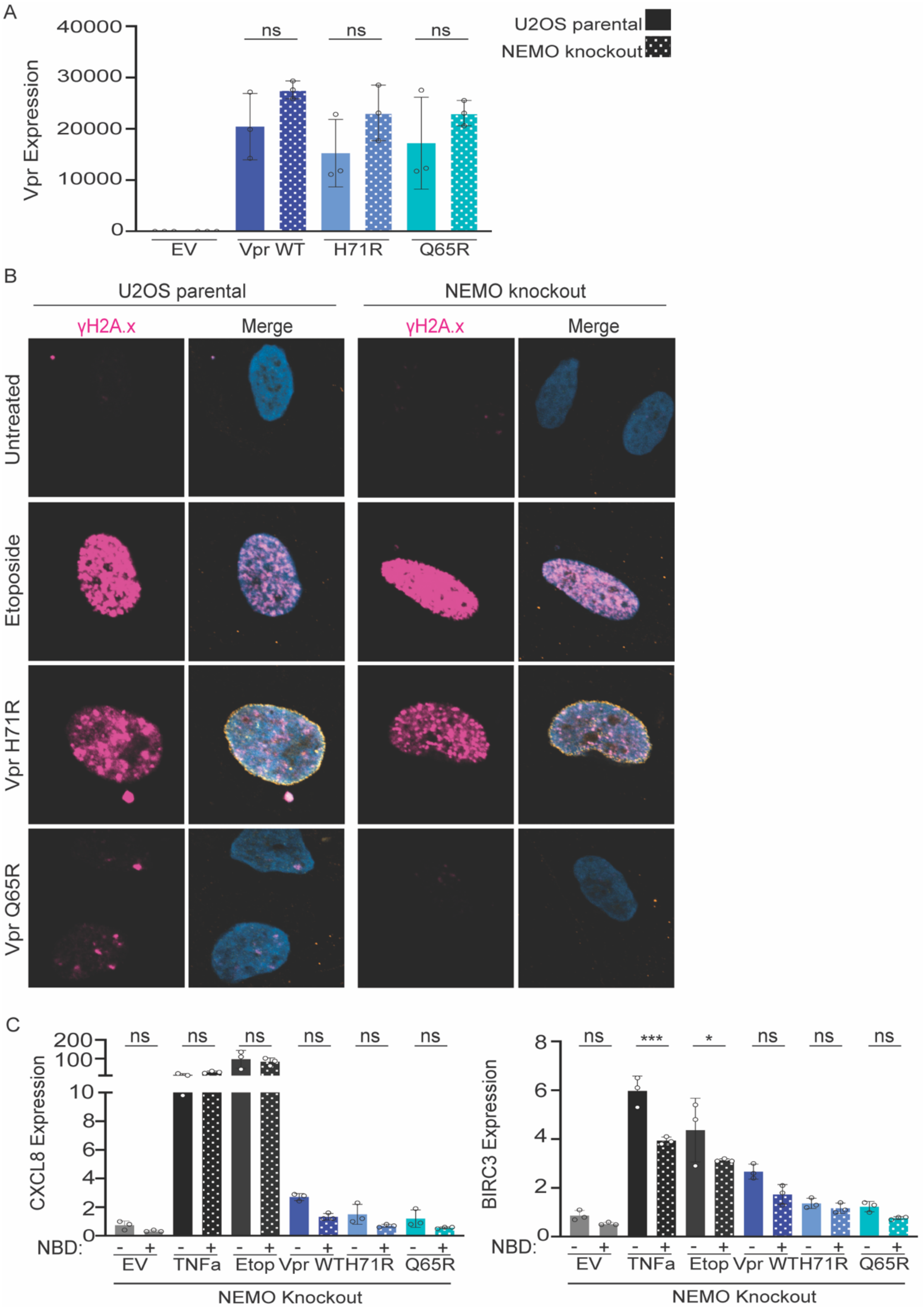
A
Vpr Expression
40000
30000
20000
10000
0
ns
ns
ns
EV
Vpr WT
H71R
Q65R
U2OS parental
NEMO knockout
B
U2OS parental
NEMO knockout
γH2A.x
Merge
γH2A.x
Merge
Untreated
Etoposide
Vpr H71R
Vpr Q65R
C
CXCL8 Expression
200
100
10
8
6
4
2
0
ns
ns
ns
ns
ns
ns
NBD:
- +
EV
TNFa
Etop
Vpr WT
H71R
Q65R
NEMO Knockout
BIRC3 Expression
8
6
4
2
0
ns
***
*
ns
ns
ns
NBD:
EV
TNFa
Etop
Vpr WT
H71R
Q65R
NEMO Knockout

Fig S5

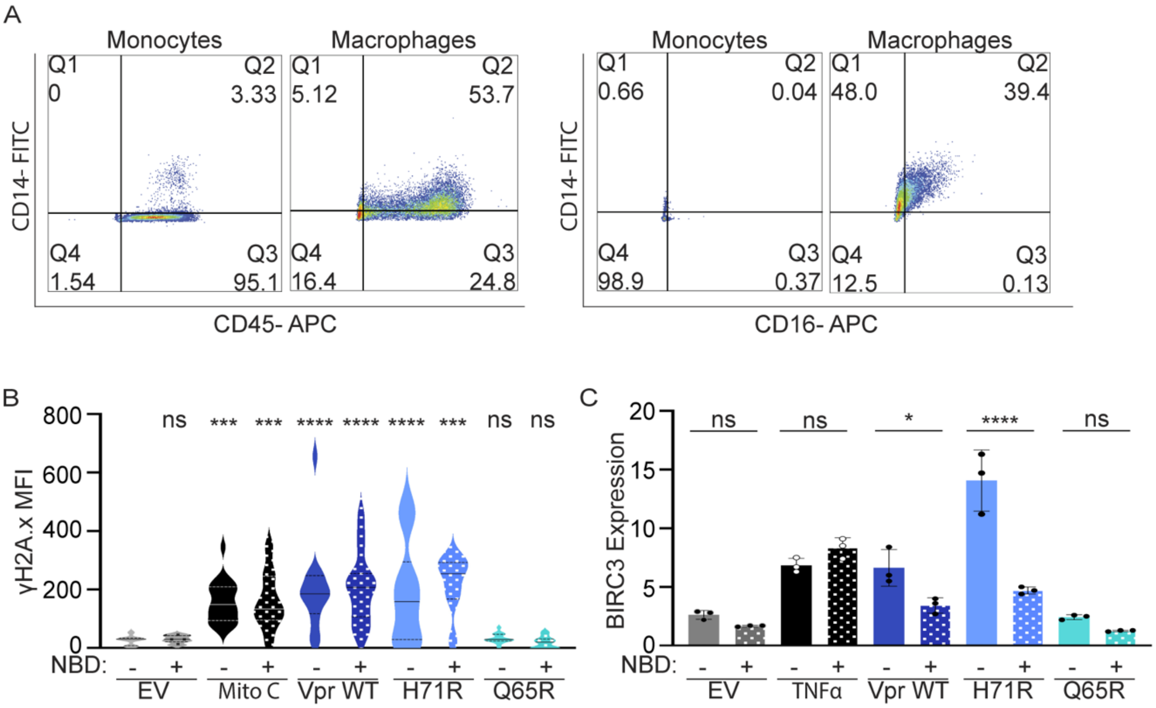

A
Monocytes
Macrophages
Q1
0
Q2
3.33
Q4
1.54
Q3
95.1
Q1
5.12
Q2
53.7
Q4
16.4
Q3
24.8
CD14- FITC
CD45- APC
Monocytes
Macrophages
Q1
0.66
Q2
0.04
Q4
98.9
Q3
0.37
Q1
48.0
Q2
39.4
Q4
12.5
Q3
0.13
CD14- FITC
CD16- APC
B
γH2A.x MFI
800
600
400
200
0
ns *** *** **** **** **** *** ns ns
NBD: - + - + - + - + - +
EV
Mito C
Vpr WT
H71R
Q65R
C
BIRC3 Expression
20
15
10
5
0
ns ns * **** ns
NBD: - + - + - + - + - +
EV
TNFα
Vpr WT
H71R
Q65R

Fig S6

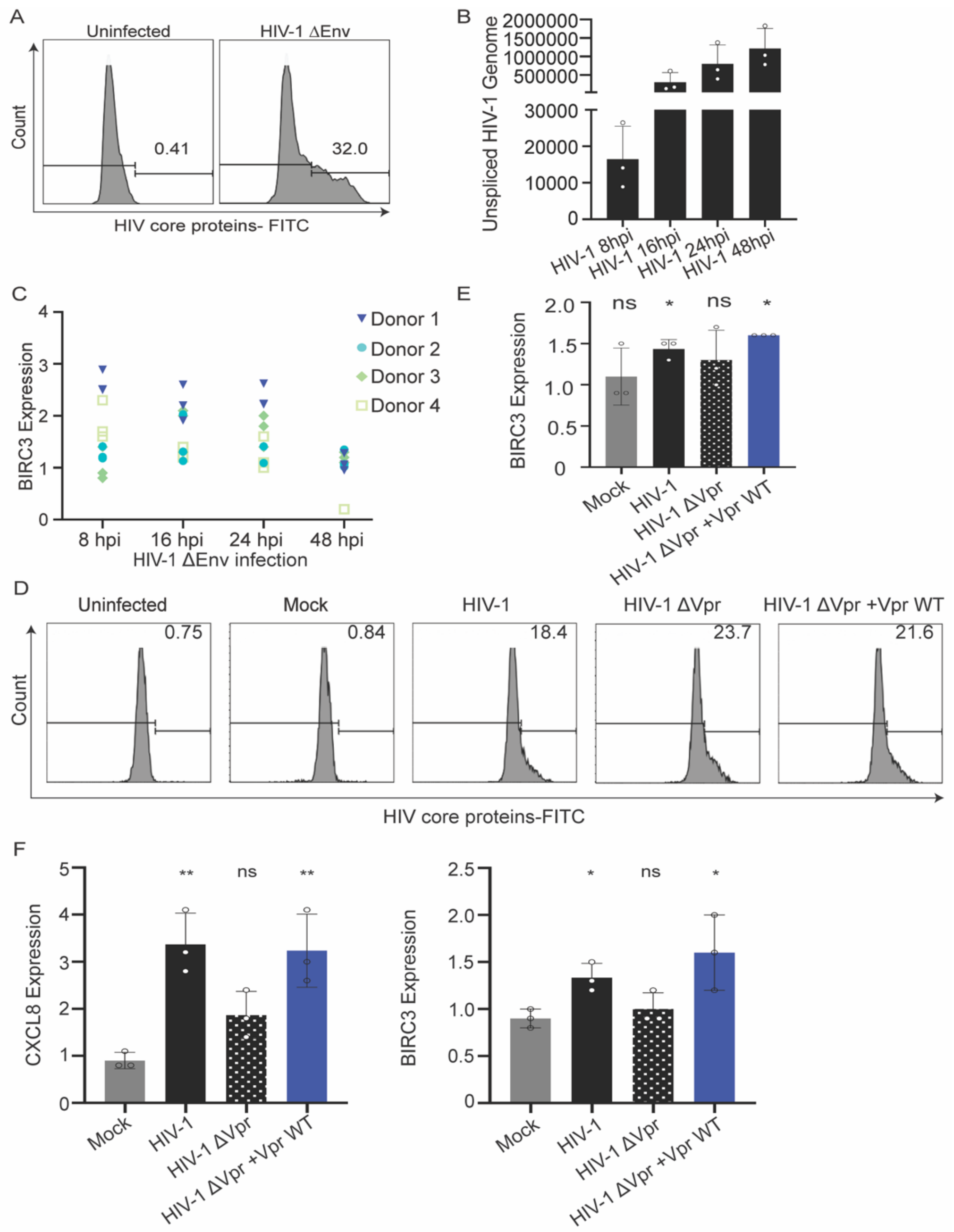

A
Uninfected
HIV-1 ΔEnv
Count
0.41
32.0
HIV core proteins- FITC
B
Unspliced HIV-1 Genome
2000000
1500000
1000000
500000
30000
20000
10000
0
HIV-1 8hpi
HIV-1 16hpi
HIV-1 24hpi
HIV-1 48hpi
C
BIRC3 Expression
Donor 1
Donor 2
Donor 3
Donor 4
8 hpi
16 hpi
24 hpi
48 hpi
HIV-1 ΔEnv infection
E
BIRC3 Expression
ns
*
ns
*
Mock
HIV-1
HIV-1 ΔVpr
HIV-1 ΔVpr +Vpr WT
D
Uninfected
Mock
HIV-1
HIV-1 ΔVpr
HIV-1 ΔVpr +Vpr WT
0.75
0.84
18.4
23.7
21.6
Count
HIV core proteins-FITC
F
CXCL8 Expression
**
ns
**
BIRC3 Expression
*
ns
*
Mock
HIV-1
HIV-1 ΔVpr
HIV-1 ΔVpr +Vpr WT

SUPPLEMENTAL FIGURE LEGENDS

Fig S1: Characterization of HIV-1 Vpr mutants S79A, H71R, and Q65R. (A) Representative western blot of 3X FLAG-Vpr WT and mutants from U2OS cells infected with rAAV expressing Vpr WT, Q65R, H71R, S79A, or empty vector (EV) (negative control) as in Fig. 1A. α-tubulin (loading control). Quantification of Vpr levels relative to corresponding α-tubulin and normalized to Vpr WT. (B) Western blot and quantification of U2OS infected with rAAV expressing empty vector or 3X FLAG-Vpr compared to U2OS infected with mock or 5,000 U/mL RT activity of HIV-1 ΔEnv expressing Vpr at 24 hours post infection. Asterisks (*) indicates nonspecific antibody band. (C) Cell cycle arrest analysis of U2OS cell treated as in Fig. 1A. Percent of events in G2 and G1 were measured using flow cytometry gating on mCherry positive cells (rAAV infection) and stained for Hoescht (DNA content). N=3, one representative experiment shown. (D) Co-immunoprecipitation against 3X FLAG-Vpr WT and mutants, probed for endogenous DCAF1, from U2OS cells infected under the same conditions as Fig. 1A. Quantification of DCAF1 immunoprecipitated with Vpr WT and mutants relative to light chain. N=3, one representative experiment shown. (E) Representative immunofluorescence images of U2OS cells infected under the same conditions as Fig. 1A. Cells were either permeabilized then fixed or fixed and then permeabilized. Vpr FLAG (orange) and DAPI (blue). Representative images taken at 24 hpi at 63x magnification. N=3, one representative experiment shown. Related to Figures 1-6.

Fig S2: Vpr alters cellular transcription through diverse mechanisms that are either dependent or independent of cell cycle arrest. (A) Gene ontology using Metascape displaying TRRUST analysis for upregulated genes. (B) Principal component analysis (PCA) of the RNA-seq data showing the relatedness of the three biological replicates compared to empty vector or untreated cells. (C) Protein-protein Interaction Enrichment Analysis using Metascape of the shared downregulated genes among Vpr WT, S79A and H71R. Related to Figure 1.

Fig S3: Vpr-induced DNA damage activates markers of ATM signaling. (A) Live-cell imaging taken on the IncuCyte of U2OS NBS1-GFP or 53PB1-GFP cells infected as in Fig. 1A for 56 hrs. Heatmap displays NBS1 and 53PB1 mean fluorescence activity (MFI) (green) as arbitrary units (AU). N=2, one representative experiment shown. (B-C) Quantification of DNA damage induced in the live-cell imaging experiment in Fig. 3A at 8, 26, 33, and 48hpi. Foci per cell and foci size were quantified using ImageJ software. Images were taken at 63x magnification using the LSM 900. N=3, one representative experiment shown with at least 50 cells per condition. Arbitrary units (AU) displayed. Asterisk indicate statistical significance compared to untreated cells (negative control) at the corresponding timepoint, as determined by one-way ANOVA test (NS, nonsignificant; * P<0.03, ** P < 0.005, *** P< 0.0003, **** P< 0.0001). Related to Figure 3.

Fig S4: Nemo knockout and NEMO-Binding inhibitor peptide (NBD) comparably inhibit Vpr-induced NF-kB activation. (A) qRT-PCR for Vpr expression of U2OS cells infected as in Fig. 4 in U2OS parental or NEMO knockout cells with technical triplicates. (B) Representative images of U2OS parental or NEMO knockout cells infected as in Fig. 1A displaying γH2A.x (magenta), 3X FLAG-Vpr (yellow), DAPI (blue). Representative images taken at 24hpi at 63x magnification. N=3, one representative experiment shown. (C) qRT-PCR of BIRC3 and CXCL8 in NEMO knockout cells in the presence of Nemo-Binding inhibitor peptide (NBD, +) or the NBD negative control peptide (-) at 36 hpi. Cells were treated under the same conditions as Fig. 4. Normalized expression to GAPDH. N=3, one representative experiment shown. Asterisk indicate statistical significance compared to NBD negative control, as determined by one-way ANOVA test (NS, nonsignificant; * P< 0.003, *** P< 0.0003). Related to Figure 4.

Fig S5: Infection of primary MDMs with VLPs packaging Vpr protein upregulates NF-κB transcription dependent on NEMO. (A) Flow cytometry plots displaying differentiation of PBMC-

derived monocytes to macrophages. Primary MDMs are CD14^{hi}, CD45+ and CD16^{hi}. N=4 one representative experiment shown. (B) Quantification of γH2A.x mean fluorescence intensity using imageJ, corresponds to Fig. 5D. (C) qRT-PCR for BIRC3 of MDMs treated with NBD inhibitor peptide as in Fig. 5C with TNFα as positive control. N=3, displaying mean of 3 separate donors. Asterisk indicate statistical significance compared to NBD negative control, as determined by one-way ANOVA test (NS, nonsignificant; * P< 0.003, *** P< 0.005, **** P< 0.0001). Related to Figure 5.

Fig S6: HIV-1 infection of primary human MDMs upregulates NF-κB transcription and is rescued by Vpr. (A) Histogram quantifying percent of infected MDMs with HIV-1 ΔEnv at 48hpi. N=4, one representative experiment shown. (B) qRT-PCR for unspliced HIV-1 RNA at 8, 16, 24 and 48hpi. N=4, one representative experiment shown. (C) qRT-PCR for BIRC3 or CXCL8 of MDMs infected with HIV-1 ΔEnv at 8, 16, 24 and 48hpi in four separate donors. N=4. (D) Histogram quantifying percent of infected MDMs with HIV-1, HIV-1ΔVpr, HIV-1ΔVpr +Vpr, mock or untreated at 48hpi. N=3, one representative experiment shown. (E) qRT-PCR for BIRC3 of MDMs infected with 5,000 U/mL RT activity of HIV-1, HIV-1 ΔVpr, HIV-1 ΔVpr +Vpr WT viruses or mock. Displaying the mean of three separate donors. Normalized expression to GAPDH. Asterisk indicate statistical significance compared to untreated negative control, as determined by Welch t-test for BIRC3 (NS, nonsignificant; * P<0.04). (F) qRT-PCR for BIRC3 or CXCL8 of MDMs infected with HIV-1, HIV-1ΔVpr, HIV-1ΔVpr +Vpr, mock at 8hpi from three separate donors. One representative experiment shown with technical triplicates. Asterisk indicate statistical significance compared to untreated control, as determined by one-way ANOVA test (NS, nonsignificant; * P< 0.003, ** P< 0.001). Related to Figure 6.

Supplemental File 1: HIV-1 Vpr upregulated genes. Upregulated genes of RNA-seq data from the top 100 differentially expressed genes from U2OS cells infected with rAAV expressing HIV-1

Vpr WT, H71R, S79A, or Q65R at 36 hours post infection. Data analyzed under stringent conditions ($Log_2(2)$ fold change p-value < 0.004 and FDR < 0.01). (Tab 1) Genes upregulated in at least one condition. (Tab 2) Shared upregulated genes among Vpr WT, Vpr S79A, and Vpr H71R. (Tab 3) Shared upregulated genes among Vpr WT and Vpr S79A. (Tab 4) Shared upregulated genes among Vpr WT and Vpr H71R. (Tab 5) Shared upregulated genes among Vpr S79A and Vpr H71R. (Tab 6) Upregulated genes by Vpr WT. (Tab 7) Upregulated genes by Vpr S79A. (Tab 8) Upregulated genes by Vpr H71R. All cutoffs, including $Log_2(1.35)$ p-value < 0.0007 and FDR < 0.01, failed to identify significant differentially expressed genes in cells expressing Vpr Q65R or empty vector. Counts per million reads mapped (CPM) Log2 fold changes were calculated relative to untreated control. Related to Fig. 1D.

Supplemental File 2: HIV-1 Vpr downregulated genes. Downregulated genes of RNA-seq data from the top 100 differentially expressed genes from U2OS cells infected with rAAV expressing HIV-1 Vpr WT, H71R, S79A, or Q65R at 36 hours post infection. Data analyzed under stringent conditions ($Log_2(2)$ fold change p-value < 0.004 and FDR < 0.01). (Tab 1) Genes downregulated in at least one condition. (Tab 2) Shared downregulated genes among Vpr WT, Vpr S79A, and Vpr H71R. (Tab 3) Shared downregulated genes among Vpr WT and Vpr S79A. (Tab 4) Shared downregulated genes among Vpr WT and Vpr H71R. (Tab 5) Shared downregulated genes among Vpr S79A and Vpr H71R. (Tab 6) Downregulated genes by Vpr WT. (Tab 7) Downregulated genes by Vpr S79A. (Tab 8) Downregulated genes by Vpr H71R. All cutoffs, including $Log_2(1.35)$ p-value < 0.0007 and FDR < 0.01, failed to identify significant differentially expressed genes in cells expressing Vpr Q65R or empty vector. Counts per million reads mapped (CPM) Log2 fold changes were calculated relative to untreated control. Related to Fig. 1D.
